# Poisoning with Dulcamara

**Published:** 1851-01

**Authors:** 


*Poisoning with Dulcamara. By Dr. Plaetschke.*—A man 40 years of age, who was using decoction of dulcamara-stalks for a cough, took, one forenoon, from three to four quarts prepared from a peck of the stalks. In the evening he was suddenly seized with numbness in his limbs, and pains in the knees and elbows, dryness of the throat, and paralysis of the tongue. These symptoms increased so much in the course of three or four hours, that he could scarcely move either his limbs or tongue. The head remained unaffected, consciousness unimpared, the pulse quiet, but small and rather hard, breathing regular, the skin cool; there was neither nausea nor vomiting. From the time which had elapsed since taking the decoction, the administration of emetics was contra-indicated; recourse was therefore had to stimulants. Camphor was given freely, and the symptoms gradually disappeared.—*Lond. Med. Gaz. from Casper's Wochenschrift.*

